# Multifaceted Characterization for the Hepatic Clearance of Graphene Oxide and Size-Related Hepatic Toxicity

**DOI:** 10.3390/molecules29061335

**Published:** 2024-03-17

**Authors:** Zongyi Su, Wei Chen, Shanshan Liang, Hao Fang, Minglu Zhang, Meng Wang, Lingna Zheng, Bing Wang, Yi Bi, Weiyue Feng

**Affiliations:** 1 School of Pharmacy, Key Laboratory of Molecular Pharmacology and Drug Evaluation (Yantai University), Ministry of Education, Collaborative Innovation Center of Advanced Drug Delivery System and Biotech Drugs in Universities of Shandong, Yantai University, Yantai 264005, China; suzy@ihep.ac.cn; 2 CAS Key Laboratory for Biomedical Effects of Nanomaterials and Nanosafety, Institute of High Energy Physics, Chinese Academy of Sciences, Beijing 100049, China; chenw@ihep.ac.cn (W.C.); liangss@ihep.ac.cn (S.L.); fanghao@ihep.ac.cn (H.F.); zhangml@ihep.ac.cn (M.Z.); wangmeng@ihep.ac.cn (M.W.); zhengln@ihep.ac.cn (L.Z.); fengwy@ihep.ac.cn (W.F.); 3 University of Chinese Academy of Sciences, Beijing 100049, China

**Keywords:** graphene oxide, hepatic clearance, multifaceted characterization

## Abstract

Understanding the final fate of nanomaterials (NMs) in the liver is crucial for their safer application. As a representative two-dimensional (2D) soft nanomaterial, graphene oxide (GO) has shown to have high potential for applications in the biomedical field, including in biosensing, drug delivery, tissue engineering, therapeutics, etc. GO has been shown to accumulate in the liver after entering the body, and thus, understanding the GO–liver interaction will facilitate the development of safer bio-applications. In this study, the hepatic clearance of two types of PEGylated GOs with different lateral sizes (*s*-GOs: ~70 nm and *l*-GOs: ~300 nm) was carefully investigated. We found that GO sheets across the hepatic sinusoidal endothelium, which then may be taken up by the hepatocytes via the Disse space. The hepatocytes may degrade GO into dot-like particles, which may be excreted via the hepatobiliary route. In combination with ICP-MS, LA-ICP-MS, and synchrotron radiation FTIR techniques, we found that more *s*-GO sheets in the liver were prone to be cleared via hepatobiliary excretion than *l*-GO sheets. A Raman imaging analysis of I_D_/I_G_ ratios further indicated that both *s*-GO and *l*-GO generated more defects in the liver. The liver microsomes may contribute to GO biotransformation into O-containing functional groups, which plays an important role in GO degradation and excretion. In particular, more small-sized GO sheets in the liver were more likely to be cleared via hepatobiliary excretion than *l*-GO sheets, and a greater clearance of *s*-GO will mitigate their hepatotoxicity. These results provide a better understanding of the hepatic clearance of soft NMs, which is important in the safer-by-design of GO.

## 1. Introduction

Understanding the final fate of nanomaterials (NMs) in the liver is crucial for their safer application. NMs have been widely used in commercial and medical products, such as cosmetics, vaccines, diagnostics, and drug carriers. Most studies have reported that exposure to NMs via various routes, such as dermal, inhalation, and ingestion, tends to result in accumulation in the liver after gaining access to the systemic circulation [1,2]. For instance, Fischer et al. found that the liver took up about 40–99% of quantum dots after administration into Sprague Dawley rats [3]. So far, most in vivo studies have focused on NMs’ accumulation at the organ level, their effects on liver structure and functions, cell-type-specific uptake, and responses [4,5,6]. However, there is still a lack of in-depth understanding of how some NMs are effectively eliminated from the liver while others achieve long-term accumulation.

The physicochemical properties of NMs determine their accumulation and clearance patterns in the liver, including their size, shape, surface coating, chemical composition, deformation, degradation, etc. [7,8,9]. For example, Chan et al. used hard NMs as a model, including quantum dots, gold nanoparticles (NPs), and silica NPs, to investigate the impact of size, composition, and surface chemistry on NMs’ sequestration and clearance [8]. This study showed that both primary rat Kupffer cells and immortalized murine macrophages preferentially uptake larger-sized NPs than smaller-sized NPs. Our previous study also demonstrated that the surface chemistry of NMs governs their sub-organ biodistribution, transfer, and clearance profiles in the liver [9]. Understanding the hepatic clearance of soft NMs (such as liposomes, micelles, and polymers) remains challenging due to their degradation properties and the lack of a simple and accessible method for their quantitative analysis [10]. Thus, multi-aspect information on the biological behavior of soft NMs in vivo is needed to understand their hepatic clearance feature.

Graphene oxide (GO) is a typical two-dimensional (2D) soft material that has shown promising applications in sensing/imaging, gene/drug delivery, cancer therapy/diagnosis, and tissue engineering/regenerative medicine [11,12]. In particular, GO NMs are among the most popular drug delivery vehicles for treating liver diseases due to their tunable chemical/physical properties [13]. The liver has a different cellular phenotype, including Kupffer cells (KCs), liver sinusoidal endothelial cells (LSECs), hepatocytes, etc., which play important roles in the uptake and clearance of NMs [8]. Previous studies have demonstrated that highly dispersed GO nanosheets may undergo biodegradation via the catalysis of myeloperoxidase secreted by phagocytic cells [14,15,16]. Our previous work has demonstrated that thin GO nanosheets, after injection, may cross the endothelial/epithelial barrier, entering the lung, liver, and renal parenchyma, and subsequently be taken up by parenchymal cells (such as alveolar macrophages, hepatocytes, renal tubular epithelial cells, etc.) [17]. The effects of the physicochemical properties of GO on the transport and clearance of GO in the lung and kidney have been systematically investigated in our previous work and by others [17,18,19]. Understanding the hepatic clearance of graphene oxide is crucial for its safe medical application [2,20]. However, so far, the final fate of GO in the liver is not yet fully understood. 

**Scheme 1:**
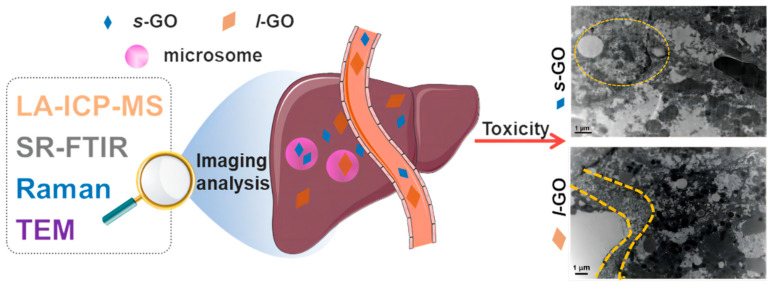
Multiscale imaging analysis for the clearance of *s*-GOs and *l*-GOs in the liver and its effects on the liver.

In this study, we prepared two sizes of PEGylated graphene oxide (GO) sheets, namely small (*s*-GO: ~70 nm) and large (*l*-GO: ~300 nm), utilizing our developed rare-earth labeling method. The hepatic accumulation and clearance of *s*-GO and *l*-GO sheets were investigated using a combination of ICP-MS, LA-ICP-MS, and synchrotron radiation FTIR techniques (Scheme 1). Furthermore, the biotransformation of GO in the liver was investigated through Raman imaging analysis of GO in the liver tissue and SRXPS analysis of the chemical speciation of GO in the liver microenvironment. Here, we present a chemical explanation for the degradation and clearance of GO in the liver. 

## 2. Results and Discussion

### 2.1. Physicochemical Properties of Graphene Oxide Nanosheets (GOs)

**Figure 1:**
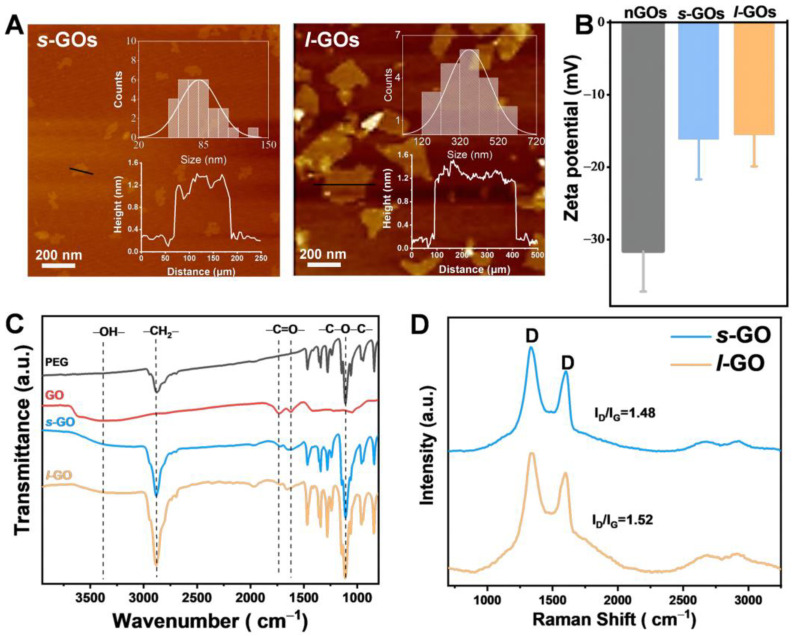
Physicochemical characterization of GOs. (**A**) AFM images of *s*-GOs and *l*-GOs. The insert figures show the thickness of GOs along the black line and the distribution of lateral dimensions of GOs. (**B**) Zeta potentials of *s*-GOs and *l*-GOs in deionized water. nGOs: non-pegylated GOs. (**C**) FTIR spectra of PEG, GO, *s*-GOs, and *l*-GOs. (**D**) Raman spectra of *s*-GOs and *l*-GOs.

The atomic force microscope (AFM) analysis reveals that the average lateral dimensions of *s*-GOs and *l*-GOs are approximately 70 nm and 300 nm, respectively, with a thickness of approximately 1 nm, corresponding to the mono-layer lamellar structure of GO (Figure 1A). Both *s*-GOs and *l*-GOs exhibit negative zeta potentials of −16.1 mV and −15.5 mV, respectively, indicating a lower absolute value of zeta potential compared to non-PEGylated GOs (−31.6 mV), which is similar to a previous study [19]. This suggests that the amine groups in PEG have neutralized some of the negatively charged carboxylic acid groups in the GOs (Figure 1B). Furthermore, the FTIR peaks of GO indicate the presence of oxygen-containing groups, such as strong bands at approximately 3378 cm^−1^ (-OH), 1616 cm^−1^ (C=O), and 1116 cm^−1^ (C-O-C), indicating that NH_2_-PEG-NH_2_ is successfully coated on the GO (Figure 1C) [20]. The typical Raman spectra of *s*-GOs and *l*-GOs are characterized by a D band at approximately 1328 cm^−1^ and a G band at about 1600 cm^−1^, with I_D_/I_G_ ratios of 1.48 and 1.52, respectively, indicating that *s*-GOs have a similar oxidation degree to *l*-GOs (Figure 1D).

To monitor the hepatic clearance of GO, a rare-earth labeling method has been employed for the analysis of GO concentration in the liver, as previously reported in a study [18]. The labeling efficiency (35.8 and 42.3 mg/g for *s*-GOs and *l*-GOs, respectively) and stability of Yb^3+^ on *s*-GOs and *l*-GOs have also been reported in a previous study [20].

### 2.2. Size-Dependent Hepatic Clearance Patterns of GO

Understanding the hepatic clearance of nanomaterials (NMs) is crucial for the development of safer NMs in the biomedical field [2]. Previous studies have reported that the unique structures and blood flow features of the liver facilitate the sequestration and accumulation of NMs, potentially leading to adverse effects in the liver [21]. The clearance patterns of hard NMs, such as Au, Ag, and QDs, have been carefully investigated [8]. In the liver, Kupffer cells, hepatic B cells, and liver sinusoidal endothelial cells may uptake these NMs. Phagocytic Kupffer cells and hepatocytes represent the two major cellular phenotypes involved in the hepatic clearance of NMs [22]. Additionally, size, as one of the most important physicochemical parameters of NMs, plays an important role in regulating target cell types and degradation pathways that interact with the liver [23]. Generally, NMs larger than approximately 200 nm are effectively cleared by Kupffer cells due to the slow blood flow in liver sinusoids, allowing sufficient time for NM uptake. The endothelium of the hepatic sinusoids is discontinuous, interspersed with pores of approximately 100–200 nm in diameter, providing an opportunity for NMs smaller than the fenestrations to cross the endothelium into the Disse space, then enter the lymphatic circulation or be taken up by hepatocytes [22].

**Figure 2:**
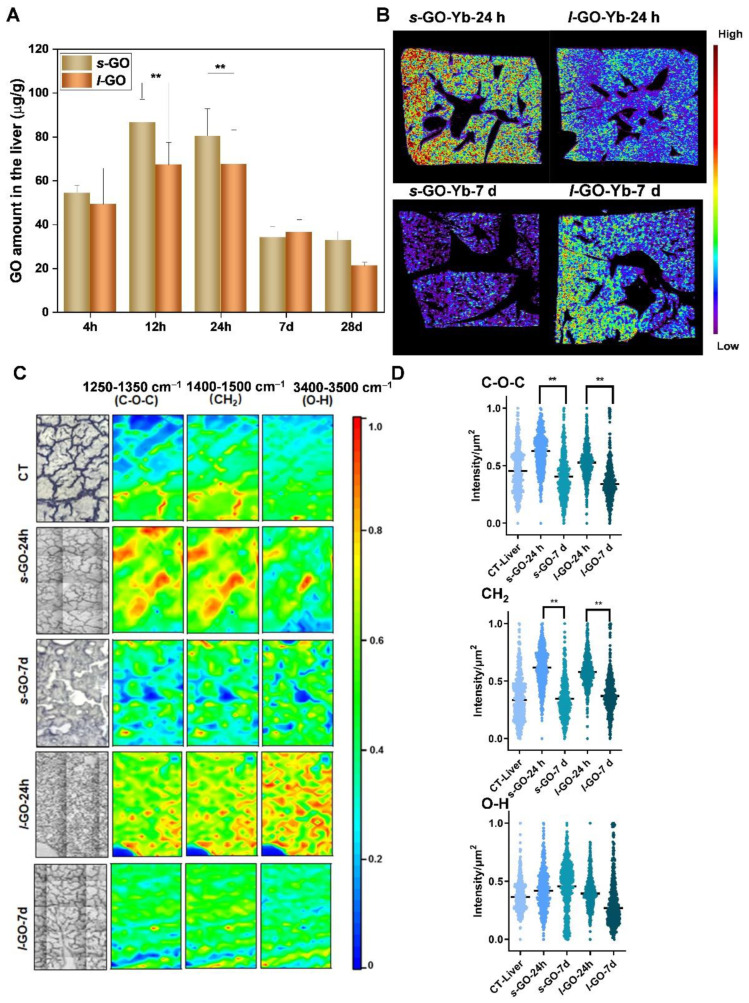
Quantitative and image analysis of deposited GOs in the liver. (**A**) Quantitative results of GOs in mice liver measured by ICP-MS. (**B**) LA-ICP-MS images of the Yb labeled GOs (*s*-GOs/Yb and *l*-GOs/Yb) in liver distribution at 24 h and 7 d post-injection. (**C**) SR-FTIR image of mouse liver tissue after treatment with *s*-GOs and *l*-GOs at 24 h and 7 d post-injection. (**D**) Quantitative analysis of the mouse liver after i.v. injection of *s*-GOs and *l*-GOs at 24 h i.v. post-injection. ** *p* < 0.01.

The clearance pattern of *s*-GOs and *l*-GOs was analyzed by detecting labeled Yb via ICP-MS. The data show that the content of *s*-GOs (54 µg/g) in the liver of *s*-GO-treated mice was slightly higher than that in *l*-GO-treated mice (49 µg/g) after 4 h of intravenous injection (Figure 2A). Both *s*-GO and *l*-GO contents in the liver show a gradual increase from 4 h to 24 h post-injection, reaching a maximum (80 µg/g for *s*-GOs and 67 µg/g for *l*-GOs) at 24 h post-injection, then gradually decreasing within 7 days after injection (34 µg/g and 36 µg/g for *s*-GOs and *l*-GOs, respectively), indicating that both *s*-GO and *l*-GO might be cleared from the liver. 

Furthermore, we used LA-ICP-MS elemental imaging technology to clearly reveal the distribution and clearance pattern of GOs in the hepatic parenchyma (Figure 2B). A comparison of the signal intensities in the tissue sections of mice 24 h after GO injection revealed that the distribution of *s*-GO in the liver was significantly higher than that of *l*-GO. At 7 d post-injection, the signals of *s*-GOs notably decreased in the hepatic parenchyma, indicating the hepatic clearance of *s*-GOs (Figure 2C). 

Similarly, synchrotron FTIR images (SR-FTIR) also demonstrated the hepatic clearance of *s*-GO and *l*-GO. The synchrotron FTIR images of the liver at 24 h and 7 d post-injection of *s*-GO and *l*-GO are shown in Figure 2D. In our previous work, the characteristic IR bands of biological tissues untreated with GO, GO material, and PEG were analyzed [18], which showed obviously distinct peaks between PEG-GO and biological tissues (including O-H stretching and C-O-C stretching); thus, the different bands of biological tissue treated with GO were rationally assigned. Specifically, the absorption features at 1437 cm^−1^ (-CH_2_ stretching) are attributed to the PEG polymer; the absorption band at 1291 cm^−1^ (C-O-C stretching) is assigned to the PEG polymer or GO; the band at 3439 cm^−1^ (O-H stretching) is assigned to GO. The quantitative analysis showed that the intensity of C-O-C and CH_2_ peaks in the liver of *s*-GO- and *l*-GO-treated mice and O-H intensity in *l*-GO-treated mice significantly decreased at 7 d post-injection compared to that at 24 h post-injection, indicating the hepatic clearance of GO, which was consistent with the results of ICP-MS and LA-ICP-MS. 

### 2.3. Hepatobiliary Excretion of s-GO and l-GO

The excretion pattern of NMs is closely related to their efficacy, circulation time, and potential side effects. It is well known that NMs are primarily excreted via the liver and kidneys [1]. Extensive urinary excretion of GO has been reported in several studies following intravenous (i.v.) injection of functionalized GO sheets in mice [20,24]. It has been observed that thin, well-dispersed GO can be excreted via the kidneys through glomerular filtration or proximal tubular secretion [20,24]. The hepatobiliary system serves as another primary route for the elimination of NMs that have not been cleared through the kidneys [25]. Compared with renal clearance, hepatobiliary excretion is generally slower, occurring over a period of hours to months [1]. In the liver, NMs that escape phagocytosis or are degraded by Kupffer cells can subsequently enter hepatocytes and potentially be excreted through bile.

**Figure 3:**
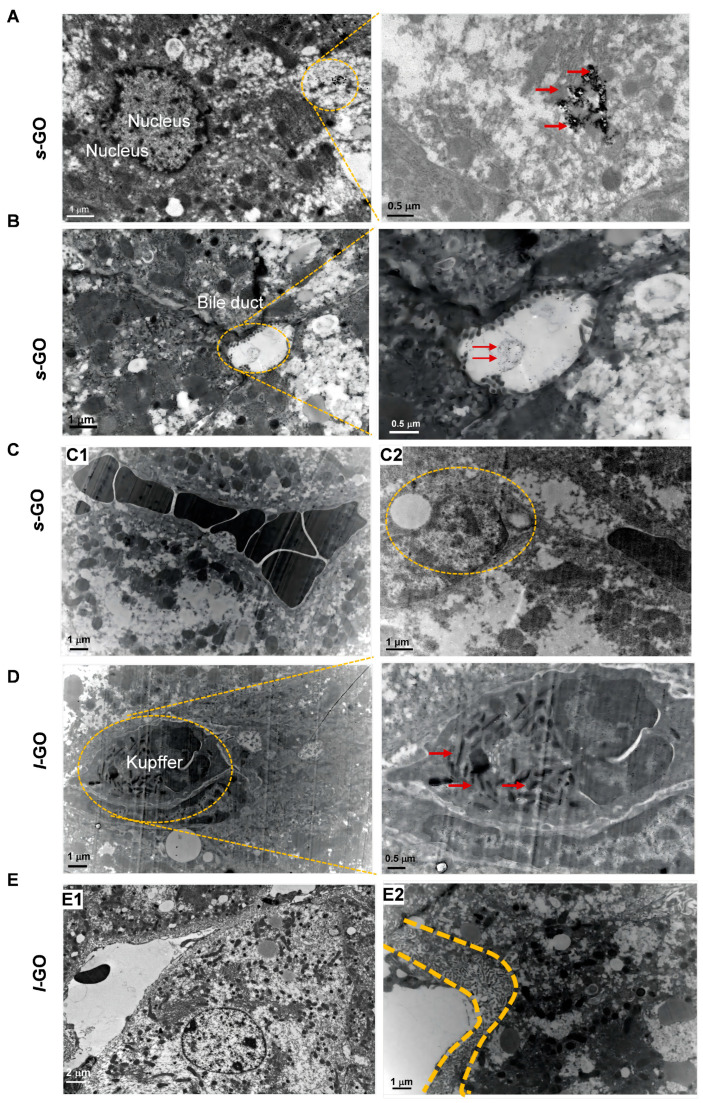
TEM images of mouse liver at 7 d after i.v. injection of *s*-GOs and *l*-GOs. A small amount of *s*-GOs (red arrows) was deposited in the cytosol of hepatocytes (**A**) and bile ducts (**B**); *s*-GOs induced sinusoidal dilation (**C1**) and enlargement of fat-storing cells, which was indicated by yellow dotted line (**C2**) (**C**); (**D**) *l*-GOs were observed in cytosol of Kupffer cells. (**E**) *l*-GOs induced sinusoidal dilation (**E1**) and collagen fibril deposition in the Disse space, which was indicated by yellow dotted line (**E2**).

In the study, transmission electron microscopy (TEM) analysis revealed that *s*-GO sheets were observed in the cytosol of hepatocytes and bile ducts at day 7 post-injection (Figure 3A,B), while *l*-GO sheets were observed in the cytosol of Kupffer cells (Figure 3D). Notably, *s*-GO sheets in the bile duct appeared as dot-like particles, suggesting possible degradation of *s*-GO within the hepatocyte. Additionally, hepatic sinusoidal dilatation (Figure 3(C1)) and enlargement of fat-storing cells (Figure 3(C2)) were observed in mice treated with *s*-GO sheets. Enlargement of the hepatic sinusoid (Figure 3(E1)) and deposition of collagen fibrils in the Disse space were also observed in mice treated with *l*-GOs (Figure 3(E2)). These findings indicate that both *s*-GO and *l*-GO may induce damage to the sinusoidal endothelium, allowing the NMs to cross the endothelium, enter the Disse space, and subsequently be taken up by hepatocytes.

**Figure 4:**
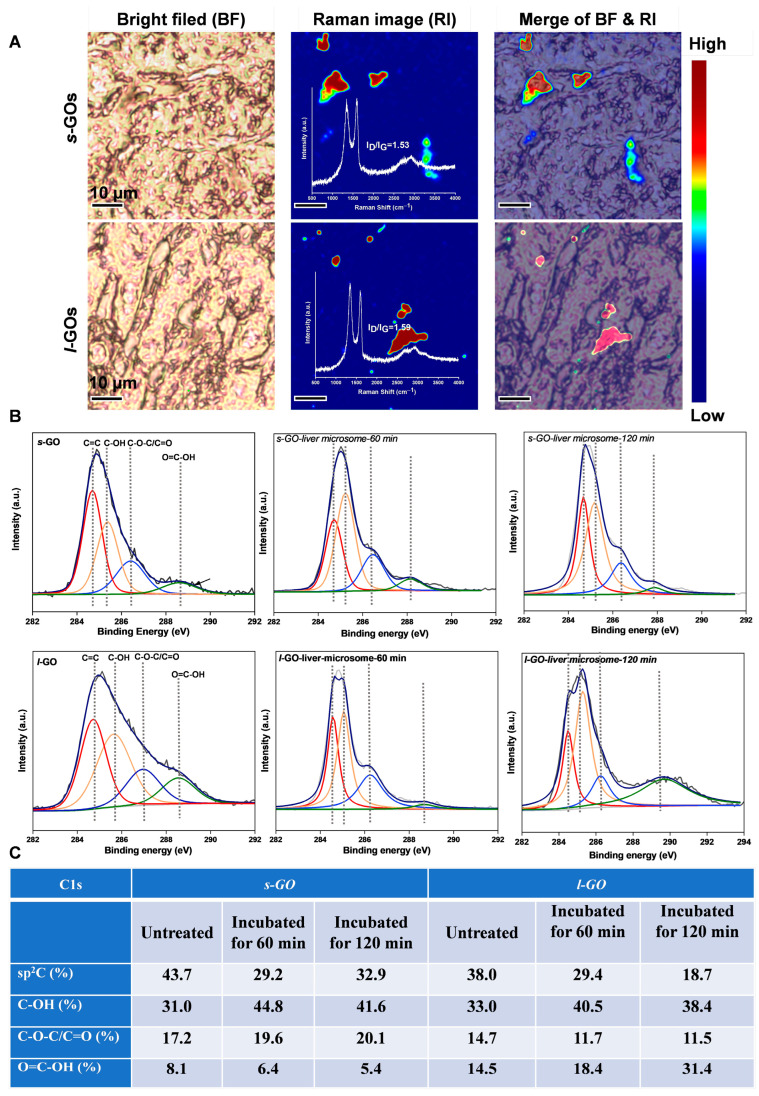
(**A**) Raman imaging analysis of *s*-GOs and *l*-GOs in the liver at day 7 after i.v. injection. From left to right: bright field (BF) image; Raman image (RI) of GOs; merged image of tissue and GOs. (**B**) SRXPS analysis of *s*-GOs and *l*-GOs at 60 min and 120 min after microsome incubation. The lines with different colors indicated different functional groups (**C**) quantitative SRXPS analysis of functional groups of *s*-GOs and *l*-GOs.

Previous studies have reported that graphene-based materials can be bio-degraded by peroxidases (such as horseradish peroxidase (HRP), myeloperoxidase, and eosinophil peroxidase) catalysis in the presence of hydrogen peroxide [14,15,16]. To demonstrate the hepatobiliary excretion process of GO, the chemical transformation of GO in the liver tissue and liver microenvironment was further investigated by Raman spectroscopy and synchrotron radiation X-ray photoelectron spectroscopy (XPS), respectively. Confocal Raman mapping (WITec, alpha 300 R, Ulm, Germany) was utilized to examine the structure of injected GO sheets in the liver sections. It was confirmed that the observed brown material in the liver (at day 7 after administration) had typical Raman fingerprints of GO (Figure 4A). Furthermore, the peak intensity ratio for the characteristic D vs. G bands (D band at ~1340 cm^−1^; G band at ~1590 cm^−1^) was calculated. The I_D_/I_G_ ratios in the liver sections of *s*-GO-treated (I_D_/I_G_ ≈ 1.53) and *l*-GO-treated (I_D_/I_G_ ≈ 1.59) mice slightly increased compared to the initial values (*s*-GO: 1.48; *l*-GO: 1.52), indicating the increased defects of GO in the liver tissue. Additionally, the chemical speciation transformation of *s*-GO and *l*-GO in the hepatocyte microenvironment was analyzed by SRXPS. Microsomes are typically used for the metabolite study because they express the major drug-metabolizing enzymes cytochrome P450 (CYP) and UDP-glucuronosyltransferase (UGT) [26]. The high-resolution XPS spectrum of C 1s shows *s*-GOs and *l*-GOs present a dominant peak at 284.6 eV, assigned to graphitic C=C species, and the other three peaks at 285.3 eV, 286.4 eV, and 288.6 eV, corresponding to C-OH, C-O-C/C=O, and O=C-OH, respectively (Figure 4B). Further quantitative analysis indicated that the C=C content of *s*-GO decreased from the initial 43.7% to 29.2% and 32.9% after 60 min and 120 min microsome treatments, respectively, and the content of C-OH increased from 31.0% to 44.8% and 41.6%, respectively, after 60 min and 120 min treatments (Figure 4C). Similarly, the C=C content of *l*-GO decreased from the initial 38.0% to 29.4% and 18.7% after 60 min and 120 min microsome treatments, respectively, and the content of C-OH increased from 33.0% to 40.5% and 38.4%, respectively. In addition, the content of O=C-OH of *l*-GO obviously increased from 14.5% to 31.4% after 120 min of treatment. A previous theoretical calculation study suggested that HRP was preferentially bound to the basal plane of GO rather than the edge, which facilitates the closer proximity of the HRP heme active site (FeO^3+^) to GO and the oxidation of the basal plane of GO [27]. Cytochrome P450 (P450, CYP) enzymes, a main component of the microsome, are adept at C=C oxygenation via hydroxylation, epoxidation, or carboxylation reactions by the formation of the active compound (FeO^3+^ or Fe^III^-O_2_^−^) involving the use of molecular oxygen [28]. Thus, the increased content of C-OH or O=C-OH in *s*-GO or *l*-GO may be related to P450-catalyzed oxidations of the GO basal plane; however, further investigation is needed. Combined with the Raman spectrum and the XPS spectrum, the defects of *s*-GO and *l*-GO in the liver section might be derived from the modification or oxidation of GO, which will facilitate the degradation of GO [16]. During this process, the graphene material may cause the dysfunction of CYP450 and trigger potential toxicity [29]. 

### 2.4. Size-Dependent Hepatic Toxic Effects of GO 

**Figure 5:**
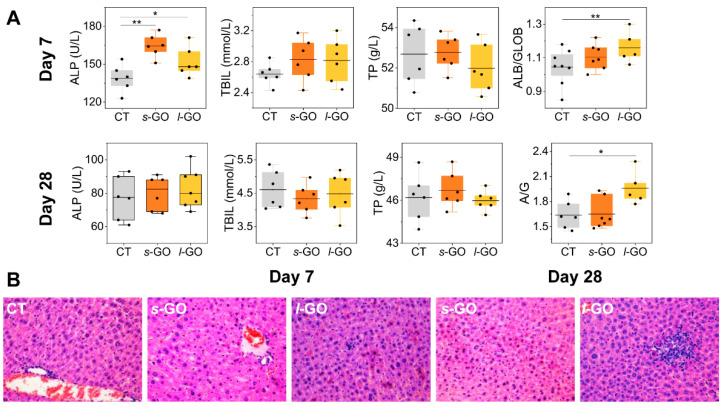
(**A**) The serum levels of liver functional biomarkers after i.v. injection of 5 mg/kg *s*-GOs and *l*-GOs to mice at day 7 and day 28 post-injection (*n* = 6). The boxes mark the intervals between the 25th and 75th percentiles; the lines inside the boxes denote medians; the dots indicate a set of measured values. (**B**) H&E staining of liver in 5 mg/mL *s*-GO-treated and *l*-GOs-treated groups. * *p* < 0.05, ** *p* < 0.01.

The serum biochemical profiles showed a significant elevation of serum alkaline phosphatase (ALP) at day 7 post-injection of *s*-GO, which returned to normal levels at day 28 post-injection (Figure 5A), indicating a stress response to the treatment. *l*-GO treatment induced a significant elevation of ALP levels and the albumin to globulin ratio (A/G) at day 7 post-injection, and the A/G ratio still remained significantly elevated at day 28, suggesting that liver function may be slightly disturbed after injection of *l*-GO. The histological changes were consistent with the serum biochemical changes (Figure 5B). A slight fatty degeneration of hepatocytes and proliferated Kupffer cells were observed at day 7 and day 28 post-injection of *s*-GO, while focal necrosis of hepatocytes within a lobule was also observed at day 7 and 28 post-injection of *l*-GO in addition to enlargement of Kupffer cells.

Previous studies have shown that unmodified GOs could induce lipid peroxidation, oxidative stress, and the secretion of proinflammatory cytokines IL-1β and TNF-α [30]. It has also been reported that tissue distribution and toxic effects are largely affected by the lateral size of the GO sheet. Larger-sized material (about 1 µm) tends to accumulate in the lungs after injection, while smaller materials accumulate mainly in the liver and spleen [31]. Furthermore, size effects of GO on hepatic impact suggested that most smaller GO sheets (lateral size ~90 nm) could be visualized inside Kupffer cells (KCs), liver sinusoidal endothelial cells (LSECs), and hepatocytes, while most larger GO sheets (lateral size ~500 nm) showed adsorption on the plasma membrane with limited cellular uptake, which elicited stronger plasma membrane lipid peroxidation, calcium flux, mitochondrial ROS generation, and NLRP3 inflammasome activation [32]. Further studies have reported that PEG modification of GO improves its biocompatibility compared to unmodified GO [33]. For instance, GO-PEG represses the progression of liver inflammation by regulating the M1/M2 polarization of Kupffer cells by inhibiting the activation of TLR3 and TLR7 in KCs [34]. In our study, *s*-GO and *l*-GO were modified by PEG; thus, *s*-GO and *l*-GO only induced slight hepatic toxicity. The lower toxicity induced by *s*-GO sheets compared to *l*-GO sheets may be closely related to hepatocyte uptake and subsequent excretion of GO. Therefore, we suggested that biomedical applications of NMs should fully consider their biological targeting, deposition, and ultimate fate.

## 3. Experimental Methods

### 3.1. Chemicals and Materials

Graphene oxide nanosheets (purity > 99%) were obtained from Jiangsu XFNANO Materials Tech. Co., Ltd., Nanjing, China. PEG-NH_2_ (molecular weight ~5 kDa) was purchased from Beijing J&K Scientific Ltd. Beijing, China. The catalysts for the amide reaction, 1-ethyl-3-(3-dimethylaminopropyl) carbodiimide (EDC) and n-hydroxysuccinimide (NHS), were purchased from Aladdin Biochemicals Tech. Co., Ltd. (Shanghai, China). 2-(4-isothiocyanatobenzyl)-1,4,7,10 tetraazacyclododecane-1,4,7,10-tetraacetic acid (DOTA-NCS) was purchased from Macrocyclics (Dallas, TX, USA). Ytterbium nitrate (Yb(NO_3_)_3_·5H_2_O, 99.9%) was bought from Strem Chemicals, Inc., Newburyport, MA, USA.

### 3.2. Preparation and Physicochemical Characterization of PEGylated s-GOs and l-GOs

The *s*-GO and *l*-GO were obtained via ultrasonication exfoliation of pristine GO for 10 h and 10 min, respectively, in an ice-water bath. Next, polyethylene glycol-modified graphene oxide was obtained by an EDC/NHS-catalyzed amide reaction at room temperature. The unreacted free PEG was removed by dialysis. 

The thickness and lateral dimensions of graphene oxide were characterized by atomic force microscopy (AFM, AFM5500, Bruker, Longwood, UK). The zeta potential of GOs in deionized water was measured using Zetasizer Nano ZS90 (MALVERN, Whitnash, UK). The characteristic Raman signals of graphene oxide were collected by a Raman spectrometer (Alpha 300 R, WITec, Ulm, Germany) equipped with a 532 nm laser. 

### 3.3. Animal Experiment

The 6-week-old male CD-1 (ICR) mice were purchased from Beijing Vital River Laboratory Experimental Animal Technology Co., Ltd. (Beijing, China). They were housed in a standard feeding environment and divided into three groups: (a) control group (100 μL PBS), (b) 5 mg/kg *s*-GO-treated group, and (c) 5 mg/kg *l*-GO-treated group.

Serum samples were collected on day 7 and day 28 after injection for biochemical analysis, including liver function. Liver tissue samples were collected at 4 h, 12 h, 24 h, 7 days, and 28 days after injection for ICP-MS analysis, LA-ICP-MS imaging, Raman imaging, Fourier transform infrared spectrometer (FT-IR) imaging, transmission electron microscopy (TEM) imaging, and hematoxylin–eosin staining (H&E).

### 3.4. Rare Earth Element Yb Label, ICP-MS Analysis, and LA-ICP-MS Elemental Imaging Analysis

Rare earth element Yb was labeled on GOs by DOTA coupling. The labeling method may be referred to in our previous published work [17]. Yb in the liver samples was quantitatively analyzed by ICP-MS (NeXION 300D, PerkinElmer, Waltham, MA, USA). The deposition of GO in liver sections was obtained by laser ablation (NWR213 laser ablation system, Elemental Scientific Lasers, Bozeman, USA) coupled to an ICP-MS system (LA-ICP-MS). Frozen tissues were sliced into 10 μm thin slices at −20 °C. Tissue sections on glass substrates were line scanned by LA-ICP-MS to obtain the distribution of GO (labeled Yb) in the liver. The scanning speed was 60 µm/sec, and the imaging spot size was 60 µm. The acquired data were analyzed with Igor Pro 6.0 software (Wavemetrics, Lake Oswego, OR, USA). The intensity of the Yb signal was transformed into the GO signal via the labeling efficiency and normalized using ^13^C as an internal standard. 

### 3.5. Confocal Raman Spectroscopy Imaging

Raman signature signals of GOs in the liver were obtained using a WITec alpha 300 R confocal Raman microscope (WITec, Germany) (532 nm laser excitation source and 600 line/mm grating spectrograph). Raman images were obtained at 0.6 × 0.6 µm^2^ pixel resolution at 500 ms/point integration time. 

### 3.6. Synchrotron Radiation-Based FT-IR Imaging

Mice were perfused with 0.9% NaCl and 4% paraformaldehyde, then the liver tissues were frozen, cut into 10 μm slices adhered to the surface of BaF_2_ slices, naturally air-dried, and then imaged by synchrotron radiation-based Fourier transform infrared (SR-FTIR) micro-spectroscopy (Shanghai Synchrotron Radiation Facility-SSRF, BL01B, Shanghai, China).

The infrared spectra were collected using a Nicolet 6700 Fourier transform infrared spectrometer (Nicolet 6700 FTIR, Thermo Fisher Scientific, Waltham, MA, USA) and a Nicolet continuous infrared microscope between 4000~800 cm^−1^ in transmission mode. Spectral imaging was performed by raster scanning of 20 × 20 μm^2^ liver sections with a spectral resolution of 4 cm^−1^. All FT-IR profiles were processed using Omnic 9.0 (Thermo Fisher Scientific Inc., Waltham, MA, USA). The presentation of test results is determined by the signal strength per unit area.

### 3.7. TEM Imaging 

TEM (JEM-1400Flash, JEOL, Tokyo, Japan) was used to observe the deposition of GOs in liver tissue and the ultrastructural changes in the liver. The liver tissues were fixed using 2.5% glutaraldehyde at 4 °C for more than 12 h and then proceeded to fixation with 1% osmium tetroxide for 3 h. Next, the sample was dehydrated, resin-coated, and cut into 70 nm slices, then stained with uranyl acetate and lead citrate. 

### 3.8. Statistical Analysis 

All mentioned data were presented as mean ± standard deviation and statistically tested by one-way ANOVA or Student’s *t*-test to compare direct differences across groups. By comparison, a *p*-value of less than 0.5 was considered to be a significant difference between different groups. Significant levels were set at * *p* < 0.05 and ** *p* < 0.01.

## 4. Conclusions

Overall, *s*-GO showed lower hepatotoxicity than *l*-GO. It is possible that GO sheets may cross the hepatic sinusoidal endothelium and then be taken up by hepatocytes via the Disse space. Hepatocytes may degrade GO into dot-like particles, which may be excreted via the hepatobiliary route. Microsomes, which possess an abundance of phase I and phase II drug-metabolizing enzymes, may catalyze oxidation, reduction, and hydrolysis reactions, contributing to the transformation of GO into O-containing functional groups, which play an important role in GO degradation and excretion. Particularly, it was observed that *s*-GO sheets in the liver were more likely to be cleared via hepatobiliary excretion than larger GO sheets, which may mitigate their hepatotoxicity. 

## Data Availability

The data that support the findings of this study are available from the corresponding author upon reasonable request.
